# Perspectives on COVID-19 testing policies and practices: a qualitative study with scientific advisors and NHS health care workers in England

**DOI:** 10.1186/s12889-021-11285-8

**Published:** 2021-06-24

**Authors:** Anne-Marie Martindale, Caitlin Pilbeam, Hayley Mableson, Sarah Tonkin-Crine, Paul Atkinson, Aleksandra Borek, Suzannah Lant, Nina Gobat, Tom Solomon, Sally Sheard

**Affiliations:** 1 Institute of Infection, Veterinary and Ecological Sciences, University of Liverpool, Liverpool, England; 2 Nuffield Department of Primary Care Health Sciences, University of Oxford, Oxford, England; 3 Institute of Population Health, University of Liverpool, Liverpool, England

**Keywords:** COVID-19, Testing, NHS, Semi-structured interviews, Health care settings, Healthcare workers, UK health policy, Pandemic

## Abstract

**Background:**

As COVID-19 death rates have risen and health-care systems have experienced increased demand, national testing strategies have come under scrutiny. Utilising qualitative interview data from a larger COVID-19 study, this paper provides insights into influences on and the enactment of national COVID-19 testing strategies for health care workers (HCWs) in English NHS settings during wave one of the COVID-19 pandemic (March–August 2020). Through the findings we aim to inform learning about COVID-19 testing policies and practices; and to inform future pandemic diagnostic preparedness.

**Methods:**

A remote qualitative, semi-structured longitudinal interview method was employed with a purposive snowball sample of senior scientific advisors to the UK Government on COVID-19, and HCWs employed in NHS primary and secondary health care settings in England. Twenty-four interviews from 13 participants were selected from the larger project dataset using a key term search, as not all of the transcripts contained references to testing. Framework analysis was informed by the non-adoption, abandonment, scale-up, spread, and sustainability of patient-facing health and care technologies implementation framework (NASSS) and by normalisation process theory (NPT).

**Results:**

Our account highlights tensions between the communication and implementation of national testing developments; scientific advisor and HCW perceptions about infectiousness; and uncertainties about the responsibility for testing and its implications at the local level.

**Conclusions:**

Consideration must be given to the implications of mass NHS staff testing, including the accuracy of information communicated to HCWs; how HCWs interpret, manage, and act on testing guidance; and the influence these have on health care organisations and services.

**Supplementary Information:**

The online version contains supplementary material available at 10.1186/s12889-021-11285-8.

## Background

On 30th January 2020 The World Health Organization (WHO) declared the novel coronavirus outbreak a public health emergency of international concern (PHEIC), its highest alert [1]. On 11th March it was declared a pandemic [1]. Five days later the WHO Director General raised concern over the lack of urgency to test, isolate and contact trace and called on member states to “*test, test, test*” [2]. The importance of ongoing diagnostic testing as part of an integrated approach to pandemic containment has been well documented, with a range of benefits including understanding infection rates, providing appropriate health care, preserving lives, preventing international spread and minimising response costs [3–5]. In a review which investigated diagnostic preparedness in outbreaks of previous WHO Blueprint pathogens Kelly-Cirino et al. [6] concurred with these sentiments, though found it could be “*technically challenging*” and time-consuming to develop, validate and implement testing strategies. Challenges noted by the authors included shortages of diagnostic materials, supply chain disruptions and limited national and local surveillance and diagnostic capacity [6].

At the start of the UK response to the COVID-19 pandemic testing, contact tracing and outbreak management in England was led by regional and local Public Health England (PHE) teams and laboratories [7]. The aim was to detect the presence of the virus in hospitalised patients and frontline NHS staff [8]. During the first week of processing (30.1.20–5.2.20) 427 COVID-19 real time polymerase chain reaction (RT-PCR) oral swabs were screened to detect the presence of genetic (RNA) virus material [9]. Briggs et al. [8] have argued that capacity for additional testing at that time was hindered by national PHE laboratory infrastructure, which had been designed to provide specialist microbiology and quality assurance testing rather than mass testing, and by the then limited availability of category three pathogen-ready laboratories. Concerns were later raised about the reliability and utility of RT-PCR tests as they returned false negatives and positives, could only detect the presence of the virus within a specific timeframe, and not whether a person was infectious or not [10]. UK based RT-PCR assays have been found to have analytical specificity and sensitivity of more than 95% in laboratory conditions, however this percentage has reportedly fallen in non-laboratory operational conditions [11]. For example, the authors of a study of 1041 COVID-19 patients in China [12] found that RT-PCR sensitivity could be as low as 59%, which could reduce the likelihood of an accurate result.

On 12th March 2020 the UK Government’s response to COVID-19 formally shifted from containment to delay [13]. Those with symptoms were advised to stay at home for 7 days and were informed “*testing for coronavirus (COVID-19) is not needed if you’re staying at home*” [14]. Four days later (2 days after the WHO’s request) the British Prime Minister announced the Government’s intention to increase testing in England and Wales to 25,000 tests a day, to enable NHS staff to *“look after everybody else with confidence that they are not transmitting the disease*”; no timelines were provided [15]. In April, The Department for Health and Social Care (DHSC) published a phased plan to “*upscale*” testing to 100,000 tests per day by the end of the month, starting with in-patients then extending to symptomatic staff. The plan relied on the extended use of RT-PCR swab and antibody tests using a combination of PHE, NHS and commercial lab partners. Secretary of State for Health and Social Care, Matt Hancock, stated that antibody tests would be useful in establishing who had already had the virus, so that people “*will know they are immune and get back to life as normal*” [16]. However, it was not clear how long antibodies remained in the body after infection [17] and the idea of immunity following Covid was disputed by the WHO during the month that the DHSC plan was launched: “*There is currently no evidence that people who have recovered from COVID-19 and have antibodies are protected from a second infection*” [18].

In May 2020, the Government’s overall response to testing was criticised by the Chair of the House of Commons Science and Technology Select Committee, Greg Clark MP. He argued that testing capacity “*had been inadequate,*” had “*not increased early enough*” and that “*capacity had driven strategy*” [19]. Ten days later on 28th May a delayed national COVID-19 test, trace and isolate service was launched. NHS Test and Trace is a cross-governmental programme housed within the DHSC managed by the Joint Biosecurity Centre (JBC, also launched in May 2020). It aims to increase access to and speed of testing for all, curb transmission through contact, and track infection rates [20]. Initially, the JBC was not fully operational, nor was the service, which led to delays in its capacity to test, trace and isolate [8, 21, 22]. Globally, by 20th May 2021 more than 164 million, 523,894 confirmed cases of COVID-19 and 3.4 million deaths had been reported to WHO [23]. As death rates have risen and health care systems have experienced increased demand and disruption [24], national testing strategies have come under further scrutiny [25]. As a National Audit Office report (11.12.20) highlighted, when moving forwards with mass testing (UK) “*government needs to learn lessons from its experiences so far*” [21]. Drawing on the results of interviews with UK scientific advisors and health care workers (HCWs) this paper provides insights into national COVID-19 testing policies and practices in English NHS health care settings during the first wave of the COVID-19 pandemic (March–August 2020). The aim is to assist with ongoing learning and to inform future pandemic diagnostic preparedness. This account will be of interest to national policymakers, regional and local public health officials, and local clinical managers.

## Methods

### Study and sample

The data utilised here are drawn from a larger, longitudinal study exploring the dynamics of UK national COVID-19 health policy advice and its impact on policy and health care workers (HCWs). The study started in March 2020. Institutional ethical approval was gained by both partners (University of Liverpool Ref: 5465, University of Oxford Ref: R69302). A purposive snowball sample in conjunction with a social media recruitment campaign was utilised for speed and convenience. We were aware that HCWs faced increased demands on their time at the start of the pandemic and were pleased to recruit those who came forward within the recruitment criteria. Those eligible for inclusion were senior scientists and advisors to the UK Government on COVID-19 response; and HCWs employed in NHS primary and secondary health care settings in England. We aimed to get a range of seniority and disciplines of HCWs. Our interviewees agreed to participate only on condition of anonymity. Preliminary contacts were instigated by the wider research team. Semi-structured qualitative interviews were conducted on average every 4 weeks with the same participants to explore dynamic developments, experiences and sense-making. Interviews containing data on testing were identified from the larger dataset by interviewers (CP, PA) and through key term transcript searches which included “test”, “RT-PCR” and “antibody”.

### Topic guides and interviews

Questions on testing arose from discussion of the interview topics. Scientific advisor topics included areas of current and future work, scientific developments, the role of advisors, collaborative working, and perceptions of challenges and successes during the pandemic (Additional file 1). Topics for HCWs included adaptations to clinical practice and roles, management of patients with COVID-19, resource availability, perceptions of infection control and risk, collaborative working and key challenges (Additional file 2). Participants were provided with approved information sheets, and verbal consent was gained prior to discussion taking place. Interviews were conducted via telephone or videoconferencing and lasted between 20 minutes and 1 hour; all were conducted by experienced social science researchers (CP, HM, PA). Interviews were audio-recorded (with participants’ permission) and transcribed verbatim by members of the research team and professional transcribers. Those quoted here have agreed to the use of their responses.

### Analysis

A content analysis framework (Additional file 3) was developed by the lead author and refined following discussion with all authors. The framework was informed by the seven domains of the NASSS framework (non-adoption, abandonment, scale-up, spread, and sustainability of patient-facing health and care technologies) to ensure that we considered our data in relation to aspects of health care implementation identified as important such as national and local context [26]. We also selected normalisation process theory (NPT) as a complementary framework to further highlight finer detail in two of the seven NASSS domains, as NPT is a social action theory, concerned with the different types of work that people do when implementing an initiative [27]. The lead author imported the transcripts into NVivo 12 Pro and used it to aid analysis. The NASSS and NPT influenced framework (Additional file 3) was used to guide preliminary coding of scientific advisors’, then HCWs’ transcripts. These codes were compared through discussion with co-authors. Through the course of discussion two key cross-cutting themes arose which required further analysis, themes which spoke to the context of a rapidly unfolding testing policy field.

## Results

This paper draws on a subset of 24 interviews discussing testing from 13 Caucasian participants (five scientific advisors and eight HCWs). The five scientific advisors were male; of the HCWs nine were female, five were male. The scientists were advising the UK Government on COVID-19 response, the need for anonymity prevents further description. The HCWs were drawn from NHS primary and secondary care settings and included nurses, a dietitian, a speech and language therapist, two physicians who were both departmental clinical directors and a GP partner. As the interviews were short, and we wanted to capture as much data as possible, participants were not asked for additional demographic details e.g. age. Two interviewees were interviewed once, the remainder, between two and five times each (March–August 2020). We identified two major cross-cutting themes: perceptions on testing strategies and implications; and policy implementation.

### Perceptions on testing strategies and implications

Scientific advisors and HCWs discussed the benefits, challenges and implications of results from different types of COVID-19 test. The HCWs were broadly positive about the role of RT-PCR and antibody testing in HCWs, both to identify current infection (RT-PCR testing) and thus avoid passing it to patients, other staff or those at home though isolation, and to provide clarity about a person’s previous COVID-19 exposure (antibody testing). However, concerns about the limitations of RT-PCR test results were raised, including accuracy, the limited period of the test’s relevance, lack of clarity about whether a HCW was infectious, and the implications of a positive test result.

*“ … the PCR test just detects the genetic material, it doesn’t tell you if the virus is viable … people can be PCR positive for a very long time so it’s causing difficulties in people returning to work, particularly health care workers, when they are PCR positive, but they are well, does that mean they are still infectious? Sometimes people are antibody positive and PCR positive so how do you interpret that?”* (Scientific advisor 4 2.7.20).

Speaking about their involvement with a research project exploring COVID infection rates through RT-PCR testing, one GP who was also a Clinical director of a primary care network (PCN) was concerned about the organisational implications of a positive test result

“ *… we might lose a third of our staff overnight because they might be positive … and that’s an ethical dilemma … so they test this week, but then what happens next week...so the risk is, it’s continual. It’s not something that was resolved by the testing*” (19.5.20).

The findings illustrate some tensions arising from the ongoing need to manage COVID related health risks and maintain health service provision with a reduced staffing complement. As the following quotes illustrate some HCWs perceived that antibody testing may address some of these difficulties, providing confirmation of previous infection with COVID-19,

“ *… you’re a health care professional working with Covid patients, you’ve had an antibody test … I think people would be reassured to know whether they had it or not, not just a swab at the time, because it means nothing, really, because I could be swabbed on a Tuesday and then I could actually have it that afternoon …* ” (Respiratory nurse ITU 11.5.20).

Another nurse reported “*feeling somehow protected*” in spite of an uncertain length of immunity and that a positive antibody result had given her “*a bit of peace of mind*” as she felt she was not spreading the virus (Specialist nurse ITU 11.6.20), while a clinical director stated, “*I’d also quite like to be positive to know that I’ve got an immune response*” (3.6.20). However, the idea that a HCW would be immune from COVID following infection was questioned, illustrating variations in perceptions of virus transmission and risk

“ *… we have no idea whether detection of antibodies will be protective in a year’s time*” (Scientific advisor 2 17.3.20).

“*I have heard so many doctors, even infectious diseases consultants sort of confidently say ‘I’m immune because I have got antibody’ and we just don’t know that*” (Scientific advisor 2 5.6.20).

Though not widely available during wave one, participants from both cohorts spoke of the positive value of having end of infectiousness testing. In March 2020 one scientific advisor spoke of the tension between its utility and limited availability.

“*In an ideal world we would have laboratory confirmation to say ‘you are no longer infectious’, or ‘we think it’s highly unlikely that you’re infectious’. That applies to discharge home as well, but we don’t have enough laboratory tests to do end of illness testing”* (Scientific advisor 2 27.3.20).

Similarly, though a hospital-based dietitian reported following the guidance and returning to work 7 days after COVID-19, she was concerned about her unknown status and potential capacity to infect.

“*When I came back after a week of Covid, my family, like all my friends … they were like ‘you are joking? You are coming back to work without a negative swab?’...and I’m like ‘yeah, that’s the rule, I’m going back to work’ … I was so careful in the department”* (Registered dietician 14.5.20).

In summary, the findings highlight doubts about whether RT-PCR and antibody tests were fit for purpose in relation to their policy goals, for example returning HCWs to work with the certainty they were not infectious.

### Policy implementation

At the national level two advisors were concerned about the lack of an integrated outbreak approach in the summer of 2020; in this case the lifting of lockdown restrictions before a fully functioning test, trace and isolate system had been operationalised as this quote illustrates:

*“ … no other country has tried to lift restrictions when it has had ten thousand new cases a day and an R0 of 0.7-1. And to have done so in a relatively haphazard way with some confused messages and therefore some confused outcomes … and as a result of that, there is now a higher risk that we will get a rebound … at the same time as you start to have the Autumn respiratory infections as well*”(Scientific advisor 3 3.6.20).

Concerns about the emergence of a second wave were realised in the latter half of 2020 [28].

At the local level concerns were expressed about a lack of consideration given to the implementation and implications of national NHS staff antibody testing roll out. This policy initiative was communicated in a letter (25.5.20) from NHS England and NHS Improvement to regional NHS directors, NHS trusts, and primary care organisations. A hospital based clinical director responsible for writing standard operating staff testing procedures raised questions about who should get the test results.

“*does it go to your GP? Does it go to occupational health or … does it go purely to the individual?*” (Clinical director 3.6.20).

Similarly a GP Clinical director of a primary care network raised the question of personal versus organizational responsibility for risk management and expressed some uncertainties about what the results would mean in practice.

“*whose responsibility is it to give them the results? What does that mean? How do you counsel them? Needs to be sorted out before you then just go and blanket test everyone”* (2.6.20).

The gap between testing advice, policy and implementation and its implications for outbreak management was also noted by one of the advisors:

“*it isn’t about the advice, it is all about the implementation and implementation is difficult, reaching out to every MP, every hospital, every manufacturer is not easy … but there has been too much of a separation of advice, lag phase, implementation and we can’t get that wrong … otherwise we will go very quickly back into a rebound*” (Scientific advisor 3 17.4.20).

Finally, HCWs having to isolate following a positive test result noted some difficulties when working remotely. Challenges included accessing work electronically, particularly patient files, and feelings of guilt for contacting work-based colleagues as teams were running at reduced capacity.

“*for 14 days I had to work from home without remote access. So I only had access to my emails, I couldn’t get remote access to the electronic medical records system, so I had to do telephone reviews or do anything to help the team in the hospital … I was feeling bad being at home, pestering my colleagues*” (Registered dietician 14.5.20).

Challenges to accessing RT-PCR tests were also noted in some settings, with significant delays for those that were delivered to homes (rather than attending testing centres) meaning HCWs were unsure if they were positive for COVID-19, and unclear on whether they should isolate or could return to work. Speaking about a colleague, one nurse reported a slow testing process:

“ *… she had to wait for the kit to be sent to her and then she had to send it back and wait for the result so that takes pretty much over a week until the test came back COVID positive*” (Specialist nurse ITU 24.7.20).

In summary, the data has illustrated that national testing policies were not sensitised to local realities.

## Discussion: beyond testing

### Summary

The rolling out of large-scale health interventions requires knowledge about how policies and practices are understood and experienced at multiple levels to ensure appropriate adoption and embedding, and reflective learning over time [26]. Our accounts of UK Government scientific advisors and NHS-based HCWs during the first wave of the pandemic in England have highlighted: tensions between the pace and scale of national testing developments and their communication and implementation; differences in perception between scientific advisors and HCWs about testing, infectiousness and risk; and uncertainties about the organisation and implications of testing at the local level.

### Comparison with existing literature and implications

The WHO’s call to instigate mass testing coincided with the UK Government’s plans to rapidly upscale RT-PCR and antibody testing [16]. The importance of diagnostic testing as part of an integrated approach to pandemic control has been widely reported, along with some of the challenges in doing so [6]. Some of these challenges in the UK have included an initial lack of national and local testing capacity and access to testing for HCWs [8, 21, 22].

Interpreting the intensity of political narratives and rapid upscaling of targets in England between March and May 2020, there is a sense that testing was perceived as something of a magic bullet which would lead the UK out of the pandemic, and that the results of testing HCWs would provide a sense of clarity and certainty about who had had the virus, who was immune and who could return to work [154 16, 20]. However, testing should be considered as one aspect of a holistic, integrated approach to pandemic management [3–6]. RT-PCR and antibody tests, and the Test, trace and isolate programme were viewed as an important contribution by NHS HCWs, though they were also unsure of their accuracy and our interviews highlight that they could misinterpret outcomes e.g. COVID immunity from antibody tests. In addition, testing coordination at the local level did not seem to be well developed during the first wave in England [22]. The results indicated a lack of preparedness within some organisations, with delays to providing local testing, uncertainty about the implications of a result and inadequate facilitation of home working for those isolating who were exposed and asymptomatic. Individually, HCWs were unsure of the accuracy and reliability of results; and demonstrated some inaccurate knowledge about the implications of a positive antibody test.

These findings undermine political confidence in the ability of RT-PCT and antibody tests to return HCWs to work without risk of spreading the infection [15, 16] and illuminate concerns raised by SAGE sub-group SPI-B in April 2020

“*PHE or DHSC, in collaboration with experts, should commence work now to mitigate the potential, misclassifications, misunderstandings and misuse of antibody testing to ensure that its potential benefits are realised with minimal harms. This will require the collation of evidence regarding the test performance in different UK populations; the development of materials in multiple formats to explain the test and its results (e.g. pre and post-testing); guidance to employers on what the test does and does not convey and the rights of all workers within exiting HSE legislation*” [29].

The study illustrates that the rolling out of mass HCW testing has wider implications and interacts with more complex lived realities than the call to test initially suggests. For example, rather than alleviating concerns, testing may exacerbate HCW COVID-19 concerns [28]. Therefore, testing policies and practices, benefits and challenges need to be made meaningful at all levels, including operationally – there needs to be more clarity and communication about who is being tested, why, what happens with the result and what this potentially means to HCWs, patients and health care services.

### Strengths and limitations

In regard to strengths, first, data were collected from the start of the first COVID-19 pandemic wave in England. Second, we conducted semi-structured interviews across multiple encounters, which enhanced rapport, led to richer exchanges and provided opportunities to probe important emerging narratives such as testing. Thirdly, the paper illuminates the perspectives and experiences of UK Government scientific advisors and HCWs across secondary and primary NHS care settings, groups which are not always considered together in publications. Limitations are that the interview topic guides did not focus on testing so some richness and opportunity for deeper exploration on these topics may have been lost; and we acknowledge the limitations of using a snowball sample. Unfortunately only the views of Caucasian HCWs are present. Additionally, the findings only speak to NHS HCWs and settings and do not shed light on views from HCWs working in private or other institutional settings. Finally, patient experiences and sense-making is missing from the account.

## Conclusions

Two months into the COVID-19 pandemic the WHO urged member states to increase testing as part of a comprehensive set of measures designed to prevent virus transmission. One year on, the National Audit Office has asked the UK Government to learn from its COVID testing experiences. At the start of the pandemic in the UK there were uncertainties and concerns about how a positive or negative test result might be interpreted or acted upon. Uncertainties remain. Reflecting on the UK’s future pandemic preparedness, we suggest that greater consideration be given to the quality of communication between national and local; to the realities of implementing mass HCW testing strategies, and to the implications of test results for staff, patients and health care services.

## Supplementary Information

**Additional file 1.**

**Additional file 2.**

**Additional file 3.**

## Data Availability

Please contact Anne-Marie Martindale a.martindale@liverpool.ac.uk to request data from this study.
