# Supplementary material for: Perspectives on COVID-19 testing policies and practices: a qualitative study with scientific advisors and NHS health care workers in England
Source: BMC Public Health. 2021 Jun 24;21:1216. doi: 10.1186/s12889-021-11285-8 (PMC8224254; doi:10.1186/s12889-021-11285-8)
Supplement: Supplementary file 1 — Additional file 1. [file 12889_2021_11285_MOESM1_ESM.docx]

**Appendix 1: Interview topic guide for the Scientific advisors**

1. What aspects of the response to the outbreak have you (personally) worked on?

2. What has gone well? What has gone less well? Why, in your view?

3. What decisions has your institution taken?

4. What motivated these?

5. What are the most important interactions you have had with other institutions?

6. How successful have they been?

7. What do you think is the biggest unresolved issue for the outbreak response in the UK at this moment?

8. Please indicate significant documents your institution has put in the public domain
