# Supplementary material for: Perspectives on COVID-19 testing policies and practices: a qualitative study with scientific advisors and NHS health care workers in England
Source: BMC Public Health. 2021 Jun 24;21:1216. doi: 10.1186/s12889-021-11285-8 (PMC8224254; doi:10.1186/s12889-021-11285-8)
Supplement: Supplementary file 2 — Additional file 2. [file 12889_2021_11285_MOESM2_ESM.docx]

**Appendix 2: Interview topic guide for the Health Care Workers**

*These questions and probes will be flexibly used to guide repeated interviews. The Topic Guide will be iteratively evolving over the course of the study, enabling researchers to capture and explore emergent themes.*

**EXPERIENCE OF CLINICAL SERVICE ADAPTATIONS AND READINESS**

1. **How is your clinical practice adapting/ preparing to manage patients with suspected COVID-19?**

- **What systems have changed to manage patient flows and anticipated surge demand? How will you triage patients (if relevant)?**
- **How confident are you that these changes will be effective? What makes you say that?**
- **What do you think the unintended consequences of these changes might be? (*Probe trade-offs*)**
- **Have any innovative changes been made in response to challenges?**
- **How are these changes being communicated to you?**

1. **In what way has your role changed as a result of these system level changes? How were you notified about these changes (if requested)?**

1. **What information or training have you received to help you provide care for patients during a coronavirus outbreak? For example, regarding infection, prevention and control, use of personal protective equipment, what to do if you have been exposed to a patient with Covid-19**

- **How helpful have you found this information/training? How confident are you that you can implement this training in your role?**
- **What guidelines (if any) are in place for healthcare workers who may have been exposed to Covid-19? Are these official guidelines?**

1. **What resources have you and your team/organisation been provided with to help deliver care to patients (during an outbreak)?**

- **Probes: Staff time? Money? Physical resources? What additional resources (if any) do you feel you need?**

**PERCEPTIONS OF RESILIENCE AND RESPONSE**

1. **How do you feel about delivering care to patients in the COVID-19 pandemic? (Probe: What makes you say that? What experience have you had previously that gives you confidence to deal with COVID-19)**

- **Prompts: How concerned are you about becoming infected with COVID-19?**
- **How confident are you that you can protect yourself from catching the virus?**
- **What concerns do you have for yourself/ your family/ your wider community about your role as a health professional working during the Covid-19 pandemic?**
- **Do you think/know you have had COVID-19? Is there local testing available?**
- **Has having the infection changed your perspective?**
- **To what extent have you noticed others treating your differently as a result of your work as a health professional who may/will treat patients with Covid-19? (*Probe stigma)***
- **Where would you go for emotional/ psychological support if you needed it in relation to providing clinical care during COVID-19?**

1. **How well do you think your team and your organisation have been able to respond to the Covid-19 pandemic to date?**

- **Probes: What has gone well/been difficult in terms of delivering care and/ or implementing changes as a result of COVID-19? How is the team working together? What has worked well/ less well in terms of providing mutual support? *(Capture stories if possible).***

1. **How have your patients responded to the coronavirus outbreak and public health advice? What concerns do you hear? Stories of things working well and less well?**
2. **How have you been making potentially difficult decisions about care pathways, such as who is eligible for critical care? How have you been managing palliative care, and/or end of life planning and decision-making?**

**DEMOGRAPHIC INFORMATION**

1. Workplace
2. Department
3. Geographic location
4. Specialism
5. Current role
6. Seniority
7. Gender
8. Family status
9. Caring responsibilities
