# Supplementary material for: Perspectives on COVID-19 testing policies and practices: a qualitative study with scientific advisors and NHS health care workers in England
Source: BMC Public Health. 2021 Jun 24;21:1216. doi: 10.1186/s12889-021-11285-8 (PMC8224254; doi:10.1186/s12889-021-11285-8)
Supplement: Supplementary file 3 — Additional file 3. [file 12889_2021_11285_MOESM3_ESM.docx]

**Appendix 3**

**Table 1 Overview of wave one COVID-19 testing analysis framework**

| **NASSS domains** | **NPT core constructs** |
| --- | --- |
| D1-The illness or condition |  |
| D2 Testing as a technology |  |
| D3 Value proposition |  |
| D4-5 Adopters/organisation | coherence (sense-making)  cognitive participation (relational work)  collective Action (operational work)  reflexive monitoring (appraisal) |
| D6 wider context | coherence (sense-making)  cognitive participation (relational work)  collective Action (operational work)  reflexive monitoring (appraisal) |
| D7 embedding and adaptation over time |  |
